# Diagnosis and treatment of neurofibromatosis type 1 with malignant transformation and multiple gastrointestinal stromal tumors: a case report and literature review

**DOI:** 10.3389/fmed.2026.1866075

**Published:** 2026-06-01

**Authors:** Huabin Wang, Jie Liu, Bin Huang, Zhengwei Lai, Yanfei Fang, Guomiao Fu

**Affiliations:** 1 Department of Gynecology, The First People’s Hospital of Xiaoshan District, Hangzhou, China; 2 Department of Pathology, The First People’s Hospital of Xiaoshan District, Hangzhou, China; 3 Department of B-Ultrasound Diagnosis, Hangzhou Xiaoshan District Chengxiang Health Service Center, Hangzhou, China

**Keywords:** diagnosis and treatment, extragastrointestinal stromal tumor (EGIST), gastrointestinal stromal tumor (GIST), KIT/PDGFRA gene, malignant peripheral nerve sheath tumor, neurofibromatosis type 1

## Abstract

A 51-year-old female patient was diagnosed with neurofibromatosis type 1 (NF1) in 2000 (21 years ago). In 2015 (6 years ago), she underwent surgical resection of a mass in her right upper limb. The tumor was pathologically confirmed as malignant peripheral nerve sheath tumor (MPNST) (considering malignant transformation of neurofibroma) and neurofibroma. In 2021, she was admitted to the hospital due to urinary urgency and abdominal mass. Transvaginal ultrasound showed multiple masses in the uterus, and leiomyoma was considered. Multiple fish-like bleeding masses on the surface of the uterus, sigmoid colon and jejunum were found during the operation, and then combined gynecological and gastrointestinal surgery was performed. Postoperative pathology and immunohistochemistry (CD117, DOG-1, CD34 positive) confirmed that all masses were gastrointestinal stromal tumors (GIST)(including uterine Extra-GIST (EGIST)), and C-kit/PDGFRA gene mutation was negative. After 4 years of follow-up, there was no recurrence or metastasis of GIST and EGIST. The patient underwent 4 operations for neurofibroma in the back (including 1 reoperation for recurrence). We report a rare case of NF1 with concurrent right upper limb MPNST, jejunal GIST, and multiple EGISTs in the uterus and sigmoid colon. Despite overlapping pathological features, uterine and gastrointestinal stromal tumors differ clinically. Surgery is the primary treatment; comprehensive preoperative imaging and postoperative pathological examination are critical to prevent misdiagnosis and optimize management.

## Background

Neurofibromatosis type 1 (NF1), also known as von Recklinghausen’s disease, is an autosomal dominant genetic disorder with an incidence of 1 in 3000–4,000 individuals in the general population (1). Malignant peripheral nerve sheath tumor (MPNST) accounts for 5–10% of all soft tissue sarcomas, and approximately 50% of MPNSTs occur in patients with NF1, making MPNST the leading cause of mortality in NF1 patients (2). Gastrointestinal stromal tumor (GIST) is the most common mesenchymal tumor of the gastrointestinal tract; it is a relatively rare disease, with an incidence of 6–15 cases per million population. The molecular pathogenesis of GIST involves abnormal activation of tyrosine kinases (3), but NF1-associated GISTs typically lack KIT/PDGFRA gene mutations (4). GISTs are common in patients with NF1, occurring in one-third of NF1 patients, with approximately 90% of NF1-associated GISTs arising in the small intestine, whereas sporadic GISTs are most frequently found in the stomach (5).

Approximately 5–10% of all GISTs arise at sites outside the gastrointestinal tract and are defined as extragastrointestinal stromal tumors (EGISTs). EGISTs can occur in the omentum, retroperitoneum, mesentery, pancreas, gallbladder, liver, spleen, urinary bladder, and prostate (6); however, reports of uterine EGISTs remain scarce (7).

The co-occurrence of MPNST + GIST + multiple EGISTs in a single NF1 patient is even rarer. Its rarity and distinctive clinical manifestations warrant this case report, which may provide valuable information and a reference for the clinical diagnosis and management of such complex cases.

## Case presentation

**Figure 1:**
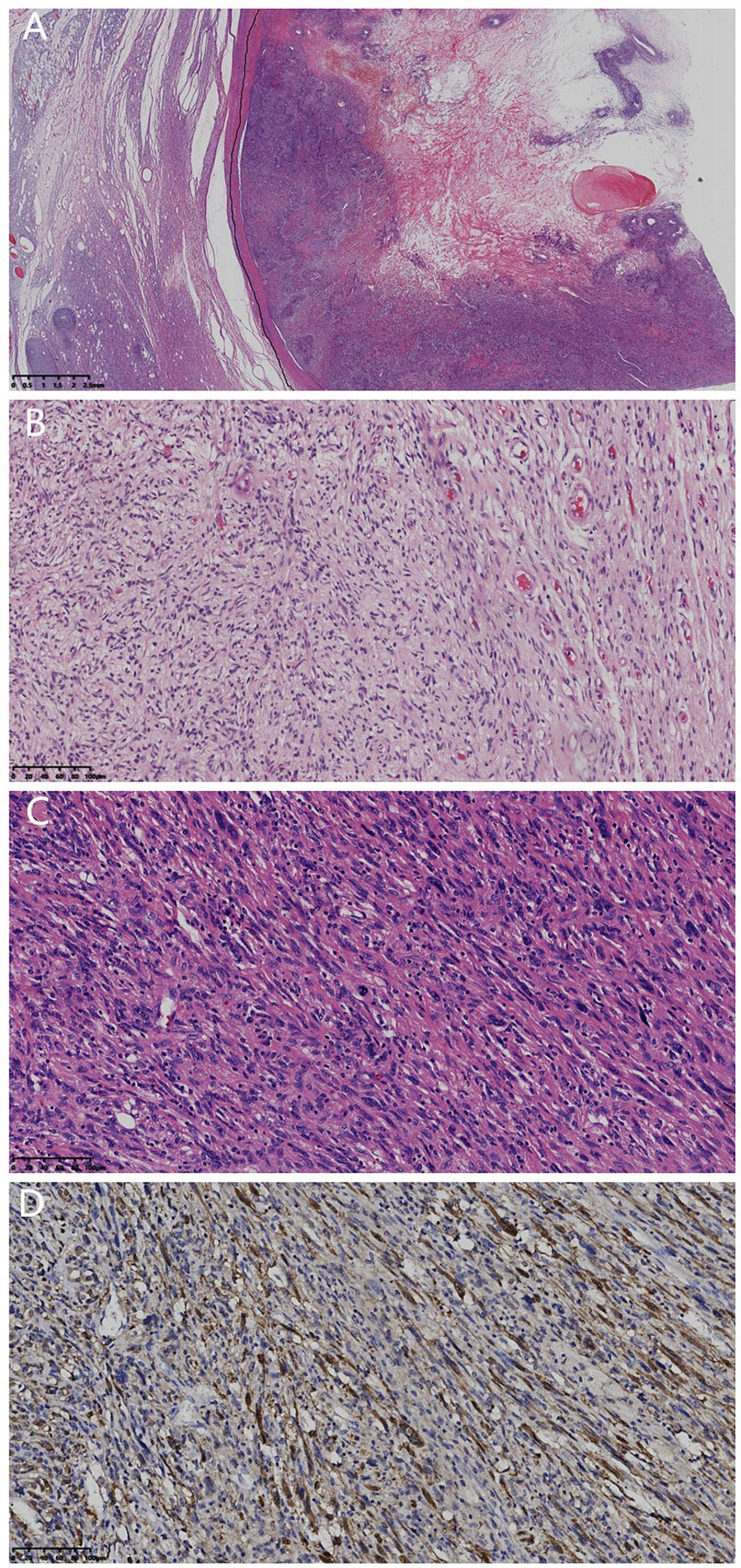
**(A)** Right upper limb tumor in 2015: NF on the left side of the black line and MPNST on the right side (HE; magnification: ×8; scale bar = 2.5 mm); **(B)** NF: spindle cells with no atypia (HE; magnification: ×200; scale bar = 100 μm); **(C)** MPNST: moderate atypia and pathological mitotic figures (HE; magnification ×200; scale bar = 100 μm); **(D)** MPNST with positive S-100 immunohistochemical staining (magnification: ×200; scale bar = 100 μm).

We present a 51-year-old female with a confirmed diagnosis of NF1 and typical clinical manifestations, including widespread café-au-lait spots (predominantly on the abdomen and back) and multiple neurofibromas; a first-degree relative (her son) was also diagnosed with NF1. The patient was initially diagnosed with NF1 in 2000 (21 years ago). In October 2015 (6 years ago), she was admitted due to two masses in the right upper limb that had been present for 4 years and had enlarged significantly with associated pain for 1 month. The two masses (10.0 × 8.0 × 8.0 cm and 3.0 × 3.0 × 2.0 cm, respectively) were surgically resected, and pathological examinations confirmed MPNST (suspected to be derived from malignant transformation of neurofibroma) and neurofibroma, respectively (Figures 1A–D). Postoperatively, the patient continued to experience needle-like pain in the right upper limb and received intensity-modulated radiation therapy (IMRT) at a total dose of 5,000 cGy in 25 fractions, which relieved the pain. In May 2018 (3 years ago), a 5.0 × 5.0 × 4.0 cm mass in the right back and lumbar region was surgically removed and pathologically diagnosed as a neurofibroma.

**Figure 2:**
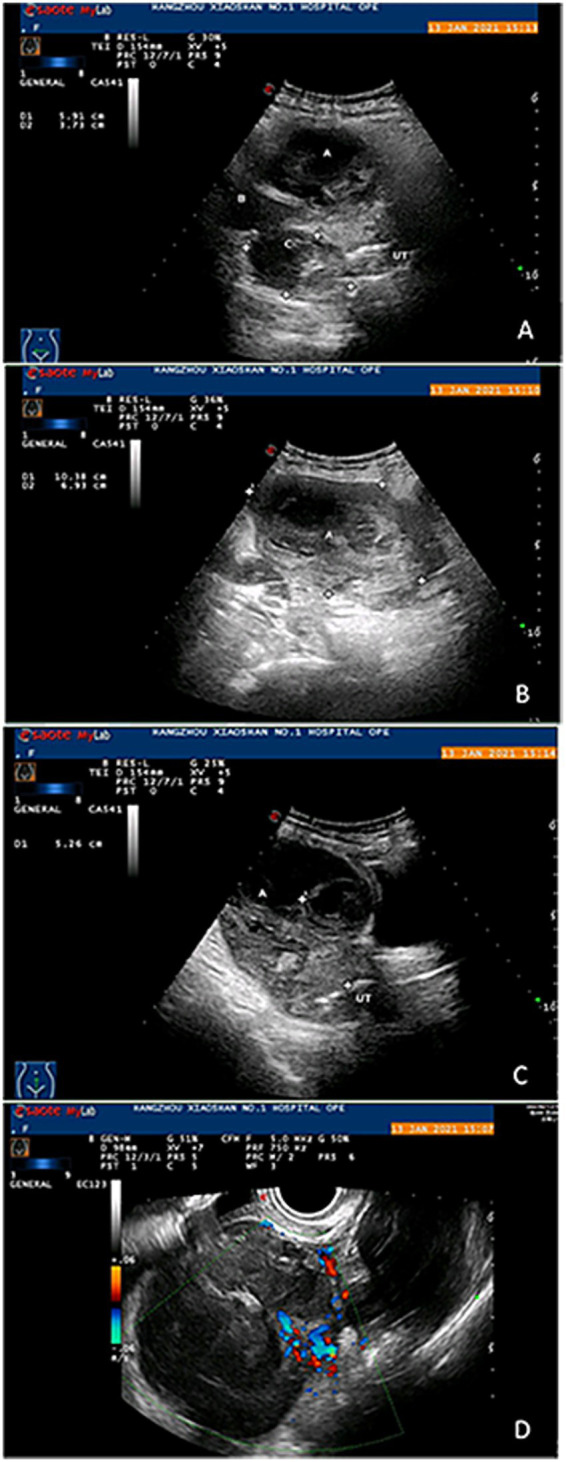
**(A)** Transvaginal ultrasound showing multiple solid uterine masses (marked A–C); **(B)** Mass A measuring 10.5 × 8.2 × 8.1 cm; **(C)** Intrauterine device in the uterine cavity with an irregular liquefaction area in mass A; **(D)** Tortuous strip blood flow signals and Adler grade II blood flow in partial tumor areas.

The patient was admitted to the Department of Gynecology with a suspected diagnosis of uterine leiomyoma, presenting with the main complaints of urinary urgency for more than 20 days and a newly detected abdominal mass for 2 days (2021). Transvaginal ultrasound showed multiple solid uterine masses (the largest measuring 10.5 × 8.2 × 8.1 cm), some with liquefaction; tortuous strip blood flow signals and Adler grade II blood flow were detected in some tumor areas. An intrauterine device was present in the uterine cavity (Figures 2A–D). The patient had moderate anemia (hemoglobin [HGB] 82 g/L; normal range: 110–150 g/L). The treating gynecologists only considered a diagnosis of uterine leiomyoma and did not perform further imaging examinations such as CT or MRI, and an open hysterectomy with bilateral salpingo-oophorectomy was planned.

### Intraoperative findings and surgical details

The operation was performed jointly by the gynecological and gastrointestinal surgery teams. The sequence of intraoperative exploration and key findings were as follows:

1. Abdominal cavity exploration: The greater omentum was densely adherent to the uterine anterior wall and bladder; the peritoneum was smooth, no masses were palpable under the diaphragm or on the liver surface, and no ascites or peritoneal nodules were present. These findings initially excluded extensive peritoneal dissemination.
2. Pelvic and uterine exploration: The uterus was enlarged to the size of a 3.5-month gestation with an irregular shape. The uterine serosal surface was covered with multiple fleshy, brittle, and easily bleeding masses, the largest (approximately 9.0 × 8.0 × 8.0 cm^3^) being located on the anterior wall. All masses were confined to the uterine serosal surface, with a clear boundary from the uterine myometrium and no evidence of myometrial invasion. The uterine fundus and posterior wall were densely adherent to a segment of the jejunum; the bilateral fallopian tubes had an indistinct morphology, and the bilateral ovaries were atrophic.
3. Intestinal system exploration: Further exploration of the small and large intestines revealed a 3.0 × 3.0 × 2.0 cm fleshy mass on the sigmoid colon serosa, which was confined to the serosal surface, separated from the intestinal muscular layer by fibrous connective tissue, and without deep intestinal wall infiltration. Three string-like masses (2.0–3.0 cm in diameter) were identified on the jejunal serosa, and further examination confirmed that these tumors had invaded the jejunal muscular layer with an intact and uninvolved mucosal surface. The remaining intestinal segments were smooth with no abnormal thickening or masses.

Surgical decision-making process: Following the identification of multiple serosal masses across different organs during surgery, the surgical team held an emergency consultation. Based on the patient’s NF1 history and the morphological and invasive features of the masses, the team highly suspected mesenchymal tumors (GIST/EGISTs). Given that the jejunal masses had invaded the muscular layer (making simple enucleation prone to residual tumor) and the presence of multifocal lesions, the surgical scope was expanded to achieve complete resection. The final surgical procedure consisted of open total hysterectomy + bilateral salpingo-oophorectomy + sigmoid colon serosal mass resection + partial omentectomy + partial jejunectomy (complete resection of the jejunal segment containing the three masses with a surgical margin of ≥2 cm from the lesion edge) + intestinal anastomosis. Intraoperative frozen section of a uterine serosal mass suggested cellular uterine leiomyoma, and the final diagnosis was confirmed by routine pathological examination and immunohistochemistry.

### Postoperative pathology and molecular detection

**Figure 3:**
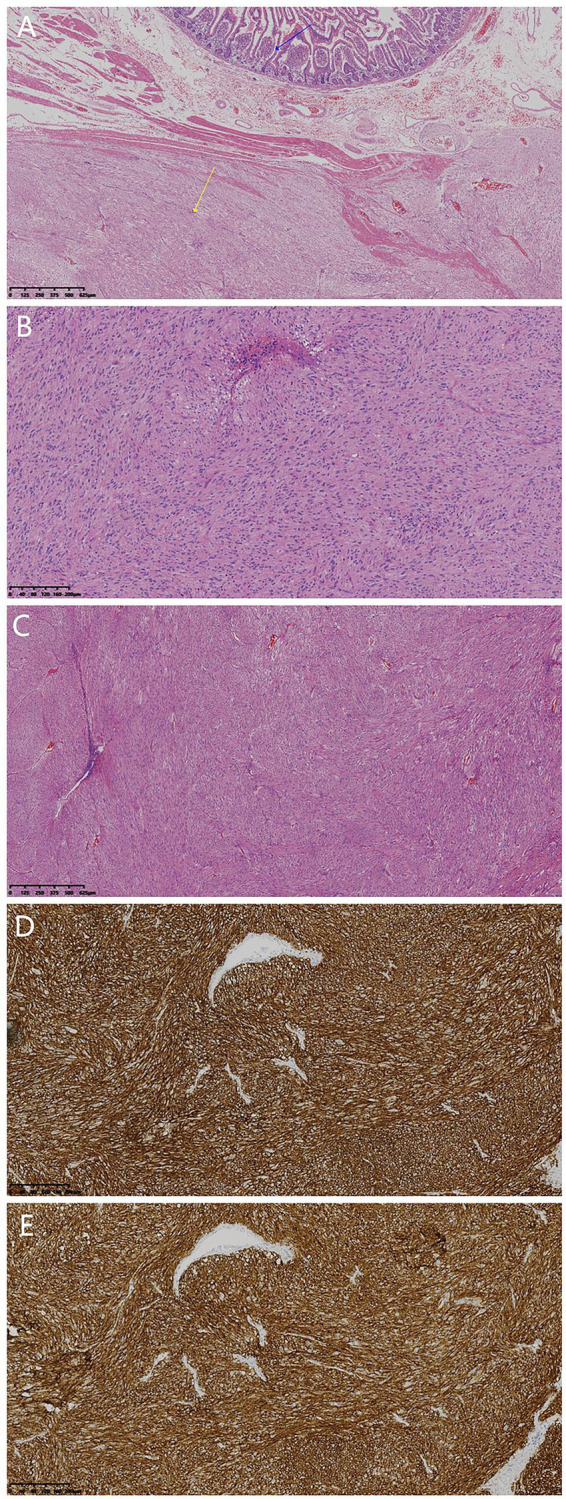
**(A)** Jejunal GIST: diffuse spindle cell proliferation with mild atypia; the tumor penetrated the serosa and invaded the jejunal muscularis propria (yellow arrow), with an intact muscularis mucosa and mucosal surface (blue arrow) (HE staining, magnification ×32; scale bar = 625 μm). **(B)** Uterine EGIST: Fascicular arrangement of spindle cells with moderate cellularity and mild nuclear atypia (HE staining, magnification ×100; scale bar = 200 μm). **(C)** Sigmoid colon EGIST: Regular spindle cell arrangement with moderate cellularity, no mitotic figures, and mild atypia (HE staining, magnification ×32; scale bar = 625 μm). **(D)** Positive CD117 immunohistochemical staining (magnification: ×100; scale bar = 200 μm). **(E)** Positive DOG1 immunohistochemical staining (magnification: ×100; scale bar = 200 μm).

Routine pathological examination (Figures 3A–C) revealed all masses to be spindle cell tumors, which were classified as GIST or EGIST based on anatomical origin and pathological characteristics.

Jejunal GIST (three lesions): Diffusely arranged spindle cells with focal increased cellular density, 1 mitotic figure per 5 mm^2^, mild cellular pleomorphism, and no necrosis. The tumor tissue penetrated the serosal layer and invaded the jejunal muscular layer; the muscularis mucosa and mucosal surface were intact, and the surgical margins were negative.

Uterine EGISTs: Spindle cells arranged in fascicles with moderate cellular density, 1 mitotic figure per 5 mm^2^, mild cellular pleomorphism, and associated liquefaction. The tumor tissue was confined to the serosal surface, separated from the uterine myometrium by fibrous connective tissue, with no evidence of myometrial invasion.

Sigmoid colon EGIST: Regularly arranged spindle cells with moderate cellular density, no mitotic figures, mild cellular pleomorphism, and no necrosis. The tumor tissue was confined to the serosal surface with a clear boundary from the intestinal muscular layer.

According to the risk classification criteria in the Chinese Guidelines for the Diagnosis and Treatment of Gastrointestinal Stromal Tumors (2023 Edition), the uterine EGISTs were classified as intermediate risk, and the jejunal GIST and sigmoid colon EGIST were classified as low risk.

### Immunohistochemical results

Antibodies were purchased from Fuzhou Maixin Biotechnology Development Co., Ltd. and Beijing Zhongshan Gene Co., Ltd. Immunohistochemical staining was performed using the EnVision two-step method. Immunohistochemical staining showed positive expression of CD117 (working fluid; Cat. No. 1910300632e), DOG-1 (working fluid; Cat. No. 1907310610c) (Figures 3D,E), and CD34 (diluent, 1:100; Cat. No. 21016826), and negative expression of SMA (diluent, 1:100; Cat. No. 21010809), CD10 (working fluid; Cat. No. 21020508), and S-100 (diluent, 1:100; Cat. No. 2012240585C8). These results were consistent with the diagnostic criteria for GIST/EGISTs and excluded other spindle cell tumors, including uterine smooth muscle tumors, uterine stromal tumors, and neurogenic tumors.

### Molecular detection results

Sanger sequencing of KIT gene (exons 9, 11, 13, 17) and PDGFRA gene (exons 12, 18) in the tumors showed no mutation (negative). There are germline mutations in the NF1 gene.

Postoperative routine blood tests confirmed correction of anemia, and the patient was discharged 12 days after surgery.

### Outcome and follow-up

#### GIST/EGIST-related follow-up

The patient was followed up jointly by gynecologists and general surgeons for 4 years (January 2021 to January 2025). During follow-up, it was recommended that physical examinations, abdominal ultrasound/CT, and routine blood tests be performed every 6 months. The patient had no symptoms such as frequent micturition, abdominal pain, or hematochezia, and no local recurrence or distant metastasis of the GIST/EGISTs was detected. Hemoglobin levels remained stable at 118–131 g/L.

Given the negative KIT/PDGFRA gene mutation results and the known biological characteristics of NF1-associated GISTs/EGISTs (which have a poor response to tyrosine kinase inhibitors such as imatinib), no targeted therapy was administered, and only regular close follow-up was conducted.

#### Neurofibroma-related follow-up

During the follow-up period, the patient underwent four surgical resections for enlarging back neurofibromas with compressive symptoms, all of which were pathologically diagnosed as benign neurofibromas. The detailed diagnosis and treatment process are summarized in Table 1.

**Table 1:** Diagnosis and treatment process of the patient.

Time (year)	Surgical procedure	Tumor size	Pathological diagnosis	Follow-up outcome
2015 (October)	Resection of 2 masses in the right upper limb	10.0 × 8.0 × 8.0 cm and 3.0 × 3.0 × 2.0 cm, respectively	MPNST and NF, respectively	No recurrence or metastasis (NR/NM)
2018 (May)	Resection of mass in the right back and lumbar region	5.0 × 5.0 × 4.0 cm	NF	NR/NM
2021 (January)	Open total hysterectomy + bilateral salpingo-oophorectomy + sigmoid colon mass resection + partial omentectomy + partial jejunectomy + intestinal anastomosis	Uterus: largest mass 9.0 × 8.0 × 8.0 cm; Sigmoid colon: 3.0 × 3.0 × 2.0 cm; Jejunum: 3 string-like masses (2.0–3.0 cm in diameter)	GIST + multiple EGISTs (KIT/PDGFRA wild-type, low + intermediate risk)	NR/NM
2021 (March)	Resection of back tumor	9.0 × 5.0 × 3.5 cm	NF	NR/NM
2023 (February)	Resection of back tumor	7.0 × 4.0 × 2.0 cm	NF	NR/NM
2023 (December)	Resection of back tumor	8.0 × 4.0 × 3.0 cm	NF	at 17 months
2025 (April)	Resection of recurrent back tumor	9.0 × 5.0 × 3.5 cm	NF	NR/NM

NF, neurofibroma; NR/NM, no recurrence or no metastasis.

## Discussion

The diagnostic criteria for NF1 are the presence of two or more of the following: 1. Cafe-au-lait spots (CALMs): ≥6 spots; The diameter was > 5 mm before puberty and > 15 mm after puberty. 2. Axillary/groin freckles: hyperpigmentation in the crease area. 3. Neurofibroma: ≥2 of any type, or 1 plexiform neurofibroma. 4. Optic nerve glioma (OPG). 5. Ocular lesions: ≥2 Lisch nodules detected by slit-lamp, or ≥2 choroidal abnormalities detected by OCT/NIR. 6. Characteristic bone lesions: sphenoid dysplasia, anterolateral bending of tibia, pseudarthrosis of long bone. 7. NF1 gene: heterozygous pathogenic variants (allele frequency 50%) were detected in normal white blood cells (8). NF1 is an autosomal dominant disorder caused by mutations in the neurofibromin 1 gene on chromosome 17. Neurofibromin negatively regulates the proto-oncogene Ras; in NF1 patients, the incidence of MPNST, GIST, optic glioma, neuroblastoma, pheochromocytoma, and breast cancer is elevated, which is likely associated with NF1 gene mutations (9). The clinical manifestations of the present patient were fully consistent with the diagnostic criteria for NF1.

Gastrointestinal stromal tumor (GIST) is the most common mesenchymal tumor of the gastrointestinal tract and originates from interstitial cells of Cajal (ICCs), the pacemaker cells for gastrointestinal peristalsis (10). Sporadic GISTs most commonly occur in the stomach (50–70%), followed by the small intestine (25–36%), colorectum and appendix (5–7%), and esophagus (1–3%) (11); approximately 90% of NF1-associated GISTs occur in the small intestine (4). Unlike sporadic GISTs, NF1-associated GISTs have a poor response to tyrosine kinase inhibitors due to the absence of c-kit or PDGFRA gene mutations (4). A systematic review including 252 GISTs in 126 NF1 patients found that NF1-associated GISTs have a distinct phenotype, including younger age at diagnosis, distal intestinal localization, smaller tumor diameter, and absence of KIT/PDGFRA mutations (5). These features are largely consistent with the present case, further supporting the avoidance of blind targeted drug therapy for such lesions.

Extragastrointestinal stromal tumors (EGISTs) are defined as GISTs arising outside the gastrointestinal tract. EGISTs were first described by Miettinen et al. (12) and account for approximately 6% of all GISTs (13). Most studies on EGISTs are case reports, and over the past 10 years, primary EGISTs have been reported in rare sites including the vocal cord, kidney, greater omentum, rectovaginal septum, prostate, vulva, pancreas, uterus, liver, and vagina (7, 14–22). Although EGISTs/GISTs share similar pathological characteristics, EGISTs have distinct clinical features. A 2023 survival analysis of EGIST patients identified age, sex, year of diagnosis, tumor grade, surgical type, and radiation therapy as independent risk factors for cancer-specific survival (CSS); EGIST patients have a poorer prognosis than GIST patients, and surgical treatment is associated with improved outcomes (23). Ambrosio et al. reported that on ultrasound, EGISTs typically present as solid, heterogeneous lobular pelvic or abdominal tumors with mixed echogenicity, possible cystic areas, rich vascularization, and no shadowing; a tumor with these features, no connection to the intestinal wall, and no origin from the uterus or adnexa is highly suggestive of an EGIST (24). In the present case, gynecological ultrasound only detected multiple uterine masses with liquefaction and Alder grade II blood flow signals, leading to a suspected diagnosis of uterine leiomyoma, which represented a deficiency in the differential diagnosis with EGISTs. EGISTs can be distinguished from intra-abdominal fibromatosis (IAF) based on 10 distinct CT criteria, including non-mesenteric localization, irregular contours, well-defined borders, heterogeneous enhancement, intralesional necrosis and vessels, absence of intralesional fat, largest diameter (LD) > 9.6 cm, LD/shortest diameter (SD) ratio >1.22, and tumor volume >603.3 cm^3^ (25).

### Diagnostic basis and differential diagnosis of uterine EGISTs in this case

The uterine lesions in this case do not support primary uterine gastrointestinal stromal tumor (EGIST), and are more likely to be secondary implantation of mesangial EGIST involving the uterine serosal, or multifocal primary pelvic/serosal EGIST involving the uterine and sigmoid serosal simultaneously.

The diagnosis was based on the following criteria: the uterine masses were confined to the serosal surface with clear boundary with the myometrium, and there was no evidence of myometrial origin and invasion, which did not meet the definition of primary uterine EGIST (7, 26). At the same time, serosal surface lesions of sigmoid colon were also found.

Identification and support points: There are two inferences: ① Multiple mesangial EGIST, transserosal implantation and secondary involvement of the uterus and sigmoid colon; ② Multiple independent primary EGIST in pelvic serosa. Moreover, the gastrointestinal stromal tumors associated with neurofibromatosis type 1 (NF1) have a tendency to occur in multiple foci, which provides a biological background for the extensive serosal multifocality of this case. It is more likely to be a multifocal lesion in the same disease spectrum rather than an unrelated independent primary tumor.

Differential diagnosis from uterine spindle cell tumors: Common uterine spindle cell tumors include cellular uterine leiomyoma, leiomyosarcoma, uterine stromal tumors, and neurogenic tumors. In this case, differential diagnosis was achieved via immunohistochemical results: SMA negativity excluded smooth muscle-derived tumors, CD10 negativity excluded stromal-derived tumors, and S-100 negativity excluded neurogenic tumors, while CD117, DOG-1, and CD34 positivity confirmed the diagnosis of EGISTs. Intraoperative frozen section suggested cellular uterine leiomyoma, and routine pathology confirmed EGISTs; this discrepancy occurred because frozen section only evaluates spindle cell morphology without immunohistochemical staining, and cellular uterine leiomyoma and EGISTs show morphological overlap under light microscopy, requiring comprehensive judgment combined with immunohistochemistry and clinical context (NF1 history, multi-organ lesions).

### Significance and interpretation of molecular detection results

In the present patient, KIT/PDGFRA gene mutations were all negative, while NF1 gene mutation was positive, a typical molecular characteristic of NF1-associated GISTs/EGISTs (4). Neurofibromin, encoded by the NF1 gene, negatively regulates the Ras/MAPK signaling pathway; when NF1 gene mutations lead to neurofibromin loss of function, the Ras pathway is constitutively activated, which may induce tumorigenesis (9). In contrast, sporadic GISTs are mostly driven by KIT/PDGFRA gene mutations that promote tumor proliferation. Therefore, tyrosine kinase inhibitors (e.g., imatinib) are highly effective for sporadic GISTs but have limited efficacy for NF1-associated GISTs/EGISTs (4). Although the jejunal GIST had invaded the muscular layer, no targeted therapy was administered due to the absence of driver gene mutations. The absence of recurrence during 4 years of follow-up further validates the clinical principle of avoiding blind targeted drug therapy for such lesions.

### Critical discussion on incomplete clinical examination

Only transvaginal ultrasound was performed prior to hysterectomy, with no further CT/MRI to evaluate the entire abdominal and pelvic cavity. This deficiency led to a missed diagnosis of multiple intestinal lesions and jejunal muscular layer invasion, and a misdiagnosis of uterine leiomyoma. This occurred because clinicians had insufficient awareness of the high tumorigenic risk in NF1 patients and limited their diagnostic thinking to common gynecological diseases, thus ignoring the propensity of NF1 patients to develop complicated multi-organ mesenchymal tumors. Additionally, the combination of non-specific symptoms (anemia + frequent micturition + abdominal mass) was not fully appreciated, leading to an incomplete comprehensive assessment of the patient’s condition.

This deficiency was not due to resource or workflow limitations but rather restricted clinical thinking, which provides an important clinical lesson: for NF1 patients presenting with abdominal/pelvic masses, unexplained anemia, or digestive/urinary symptoms (regardless of the initial department of admission), enhanced abdominal/pelvic CT or MRI should be performed to assess the extent of lesions (26), multi-organ involvement, and depth of intestinal wall invasion. For patients with suspected intestinal involvement, colonoscopy should be performed to evaluate mucosal involvement, to avoid misdiagnosis and missed diagnosis due to limited imaging examinations.

### Supplementation and comparison of the literature review

Reports of uterine EGISTs are scarce, and most are sporadic primary lesions, which differ significantly from the NF1-associated, multi-organ involved EGISTs in the present case. We compared the present case with recently reported cases of uterine/gynecological EGISTs (Table 2) to highlight its rarity and uniqueness.

**Table 2:** Comparison of clinicopathological features of uterine EGISTs.

Comparison indicators	Metachronous uterine EGIST [Ref. (7)]	Primary uterine GIST [Ref. (27)]	This case (NF1-associated multiple EGISTs/GIST)
Basic patient information	76-year-old female, no NF1	77-year-old female, no NF1	51-year-old female, diagnosed with NF1
Initial symptoms	Perineal pain, dyschezia, huge protruding lesion at vaginal introitus	Sudden severe lower abdominal pain (tumor rupture and bleeding)	Urinary urgency >20 days, abdominal mass detected for 2 days
Tumor location	Uterine body, invading vagina, densely adherent to upper rectum and sigmoid colon	Originated from uterine myometrium, no anatomical connection to gastrointestinal tract, clear boundary with uterine myometrium	Uterine serosa (multiple), sigmoid colon serosa, jejunal serosa (3 lesions); jejunal GIST invaded muscular layer, uterine/ sigmoid colon EGISTs confined to serosa
Tumor size	Pelvic tumor 10 × 10 × 9.5 cm	12 × 10 × 8.5 cm (gross specimen)	Largest uterine EGIST: 9.0 × 8.0 × 8.0 cm; sigmoid colon EGIST: 3.0 × 3.0 × 2.0 cm; jejunal GIST: 2.0–3.0 cm (3 string-like lesions)
Gross pathological features	Giant solid tumor, dense adhesion to surrounding tissues	Ruptured uterine mass, section not clearly described, clear boundary with myometrium microscopically	All masses fleshy, brittle, easily bleeding; uterine/sigmoid colon EGISTs confined to serosa (clear boundary with muscular layer); jejunal GIST penetrated serosa and invaded muscular layer
Histological type	Spindle cell tumor, no clear description of necrosis/hemorrhage	Predominantly spindle cell proliferation, minimal atypia, no obvious necrosis, tumor rupture	Spindle cell tumor; uterine EGISTs: moderate cellular density; jejunal GIST: focal increased cellular density; all lesions with mild pleomorphism and no obvious necrosis
Mitotic figures	<5 per 50 high-power fields (HPF)	4 per 50 HPF	Uterine EGIST: 1 per 5 mm^2^; sigmoid colon EGIST: 0 per 5 mm^2^; jejunal GIST: 1 per 5 mm^2^
Immunohistochemical results	CD34(+), CD117(+), c-KIT(+), SMA(+); DOG1/S-100 not mentioned	CD117(+), CD34(+), Desmin(−); DOG1 not detected	CD117(+), DOG1(+), CD34(+); SMA(−), S-100(−)
Ki-67 proliferation index	Not clearly mentioned	Not mentioned	Not clearly mentioned
Molecular detection results	No c-kit/PDGFRA mutation detection	KIT gene exon 11 internal tandem duplication mutation	KIT (exons 9,11,13,17)/PDGFRA (exons12,18) all were not mutated. Germline mutations in the NF1 gene.
Risk classification	Intermediate risk	Intermediate risk (tumor >10 cm, mitotic count 4/50 HPF)	Low + intermediate risk (jejunal GIST/sigmoid colon EGIST: low; uterine EGISTs: intermediate)
Surgical method	Exploratory laparotomy + en bloc multi-organ resection + pull-through coloanal anastomosis	Total hysterectomy + bilateral salpingo-oophorectomy + abdominal wall nodule excision	Open total hysterectomy + bilateral salpingo-oophorectomy + sigmoid colon serosal mass resection + partial omentectomy + partial jejunectomy + intestinal anastomosis
Postoperative treatment	Imatinib adjuvant targeted therapy	Not mentioned (patient died of underlying disease deterioration postoperatively)	Only regular follow-up (no targeted therapy)
Follow-up results	20 months postoperatively, disease-free survival, good treatment tolerance	Deteriorated and died the day after surgery	48 months follow-up: no recurrence/metastasis of GIST/EGISTs; 4 surgeries for back neurofibromas (1 for recurrence)

As shown in Table 2, this case describes an NF1 patient complicated with MPNST, jejunal GIST, and multiple uterine and sigmoid colon EGISTs, with the unique features of wild-type KIT/PDGFRA, multi-organ serosal involvement, and jejunal GIST with muscular layer invasion, which further enriches the clinical spectrum of GISTs/EGISTs.

## Summary

We present a rare case of a 51-year-old female with NF1 complicated by right upper limb MPNST, jejunal GIST, and multiple EGISTs of the uterus and sigmoid colon. She underwent combined multi-organ surgical resection for complete lesion removal without postoperative targeted therapy. No recurrence or metastasis of GIST/EGISTs was noted during 4-year follow-up. This case summarizes the diagnostic key points of these rare lesions, underscores the limitations of incomplete preoperative imaging and the necessity of thorough evaluation for NF1 patients with abdominopelvic masses, expands the clinical spectrum of NF1-associated tumors, and offers valuable evidence for the diagnosis, treatment and mechanistic research of similar cases.

## Data Availability

The data analyzed in this study was obtained from Hangzhou ADICON Clinical Laboratories Co., Ltd., Zhejiang, China. The following restrictions apply: Data are not publicly available due to patient privacy and ethical constraints. Requests to access these datasets should be directed to Cuilian Sun, cuilian.sun@adicon.com.cn.
